# Effect of Edible Coatings of Cassava Starch Incorporated with Clove and Cinnamon Essential Oils on the Shelf Life of Papaya

**DOI:** 10.3390/membranes13090772

**Published:** 2023-08-31

**Authors:** Allisson do Nascimento, Letícia Crestan Toneto, Bárbara Morandi Lepaus, Bárbara Santos Valiati, Leonardo Faria-Silva, Jackline Freitas Brilhante de São José

**Affiliations:** 1 Graduation in Nutrition, Department of Integrated Health Education, Federal University of Espírito Santo, Vitória 29040-090, ES, Brazil; 2 Postgraduation Program in Nutrition and Health, Department of Integrated Health Education, Federal University of Espírito Santo, Vitória 29040-090, ES, Brazil; barbaralepaus@hotmail.com (B.M.L.); barbara_30valiati@hotmail.com (B.S.V.); 3 Espírito Santo Technology Foundation, Vitória 29047-105, ES, Brazil; fariasilva.leonardo@gmail.com; 4 Department of Integrated Health Education, Federal University of Espírito Santo, Vitória 29040-090, ES, Brazil

**Keywords:** post-harvest, non-biodegradable packaging, edible film, food technology

## Abstract

Applying edible coatings added with plant essential oils is a strategy used to delay ripening processes in climacteric fruits such as papaya. Formulations comprising 3% or 4% cassava starch (*w*/*v*), added with clove or cinnamon essential oils (2 mL/L), were tested for microbial inhibition (in vitro) purposes. Moreover, these fruits’ physicochemical and microbiological aspects were assessed at 25 °C, for 12 days. Slight variations in pH and Brix values were observed during storage. On the other hand, there were no significant variations in carotenoid contents over storage time. The papaya fruits’ coating contributed to reducing their weight loss from 40.66% (uncoated sample) to 24.10% on the 12th storage day, as well as delayed changes often observed during the ripening process. The 4% cassava starch coatings added with essential oils were more efficient in reducing microbiological levels. The herein proposed treatments reduced aerobic mesophilic bacteria, as well as molds and yeast counts, by 1.48 and 1.95 log CFU/g, on average, respectively, in comparison to the control sample. The assessed microorganism counts were higher in the uncoated sample than in the coated papaya fruits, after 12 days of storage. Thus, the tested coatings can potentially delay the emergence of post-harvest changes; consequently, they can help improve the quality of papaya fruits and extend their shelf life.

## 1. Introduction

Papaya (*Carica papaya* L.) is one of the fruits mostly grown in tropical and subtropical regions worldwide [1]. Brazil is one of the largest papaya producers and exporters, and one of those capable of meeting European countries’ demands for them [2]. The papaya fruit’s popularity is justified by its sensory attributes [3], as well as by its nutritional components, such as fiber, vitamins, and minerals [4]. However, this fruit can undergo post-harvest changes, such as enzymatic deterioration (softening) [5] and fungal decay [6,7,8], which, in turn, can reduce its shelf life, as well as influence its transportation, distribution, and trading [5,7].

Post-harvest losses can influence the quality and quantity of produced fruits; moreover, they can take place throughout the handling, storage, and packaging processes in the fresh-cutting and processing industries [9]. Consumers constantly demand safer technologies and products, which has an impacts on the food industry [10]. Therefore, it is essential to adopt strategies focused on minimizing quality losses without harming consumers’ health—as happens with fungicides [6,11]—to always preserve these products’ natural features [12,13].

Edible coatings and films are new packaging materials and are considered a trending topic. They are some of the many alternatives investigated to enhance fruit quality during storage time. Coatings can comprise biological or chemical materials applied on the surface of food products to avoid gas exchange and to delay the ripening process [9]. Several polysaccharides (e.g., alginate, chitosan, cellulose, pectin, and starch) and proteins (e.g., whey and casein) can be used as edible coating components [14,15,16,17]. These types of substances could be obtained from agro-industrial by-products or waste. So, this can expand their value and contribute to economic and environmental benefits [16].

Starch is a naturally high-molecular-weight polysaccharide presenting low cost, low degradability, and good biocompatibility [18]. Cassava (*Manihot esculenta* Crantz) is one of the oldest plant species grown in Brazil, where it is often used to produce starch for cooking purposes, besides playing an important part in the dietary habits of different segments of the Brazilian population [19].

Besides presenting well-known polysaccharide-based coating features, such as selective gas transfer and barrier properties [20], cassava starch (CS) is isotropic, colorless, non-toxic, and inexpensive [21], as well as resistant to heat and humidity [22]. However, the formulation of a CS-based coating has limitations due to its hydrophilic nature and lack of antioxidant and antimicrobial properties [23,24].

Enriching CS-based coating with natural antioxidant substances, such as plant essential oils (EOs), can help increase the gel’s antimicrobial effect and prolong fruits’ shelf life [23,25,26,27]. EOs are aromatic products extracted from different plant parts, such as bark (e.g., cinnamon) and flower buds (e.g., carnation) [28]. These compounds can be used to slow down deterioration processes and to increase plant products’ shelf life because they have antimicrobial activity [29]. In addition, they have antioxidant properties enabled by tocopherols, polyphenols, and β-carotene found in them [30].

Positive effects on quality and preservation parameters have been associated with papaya fruits coated with different formulations, such as chitosan and Mentha EO [8], chitosan and *Cymbopogon citratus* EO [31], aloe vera [6,7], carboxymethyl cellulose added with different EOs [32], and potato starch [33]. Moreover, CS coatings added with salicylic acid [34] and clove EO [12,35] have also shown the potential to be used in papaya preservation processes.

Clove and cinnamon EOs have a broad antimicrobial spectrum. Thus, these oils can be used in association with coatings to obtain higher-quality products [36,37]. It is important to emphasize that because EOs are aromatic compounds, they must be compatible with the food matrix they will be used in to avoid a negative impact on sensory features, such as flavor and aroma [29]. The U.S. Food and Drug Administration considers oregano, clove, basil, thyme, and cinnamon EOs to have GRAS status [29]. 

The formulation of edible coatings featured by polysaccharides associated with EOs appears to have great application potential for fruit preservation purposes. However, the CS–EOs combination should be widely explored. Therefore, given the consequences of post-harvest losses and the requirement to use more natural methods with lower environmental impact, it is necessary to evaluate edible coatings added with EOs as alternative procedures adopted for food preservation purposes. Then, we herein propose a practical way to enable the post-harvest conservation of fresh papaya. The present study aimed to assess the impacts of using edible coatings, based on CS associated with clove EO (CLO) and cinnamon EO (CIN), on the physicochemical and microbiological features of whole fresh papaya fruits, during storage.

## 2. Materials and Methods

### 2.1. Obtaining Fruit

**Figure 1:**
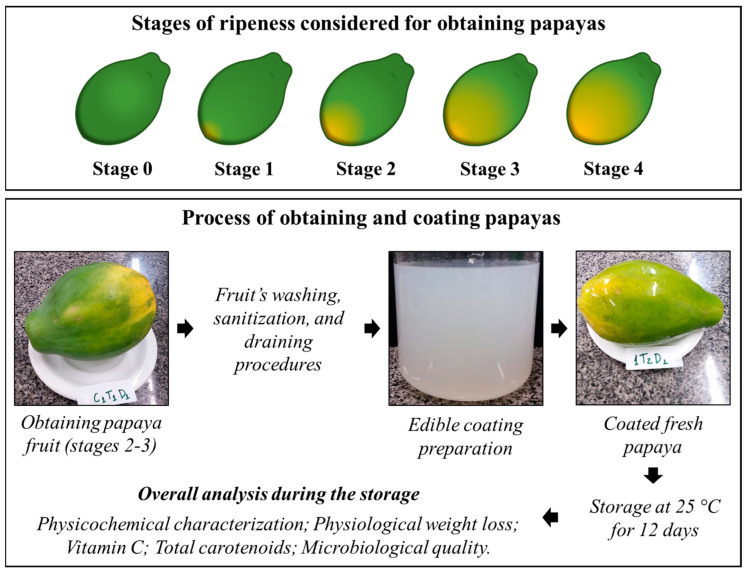
Flowchart of papaya obtainment and coating processes and summary of analyses carried out during storage time.

Papaya fruits (*Carica papaya* L.-Hawaiian variety) were purchased in the local retail market (Vitória City, Espírito Santo State, Brazil), at ripening stages 2 and 3 (Figure 1). The aforementioned stages were defined based on the fruits’ skin color, as follows: Stage 0 = fully-green fruits; Stage 1 = fruits at color break stage, with up to 15% yellow skin; Stage 2 = quarter-ripe fruits, with up to 25% yellow skin; Stage 3 = half-ripe fruits, with up to 50% yellow skin; and Stage 4 = ¾-ripe fruits, with up to 75% yellow skin. Fruits were selected, and those presenting mechanical injuries were discarded. Next, they were sanitized with 200 mg/L sodium hypochlorite solution (Nippoclor^®^, Indaiatuba, São Paulo, Brazil) for 15 min.

### 2.2. Edible Coating Preparation and Fresh Papaya Coating

Preliminary tests were carried out with different CS concentrations to assess CS (Amafil^®^, Cianorte, Paraná, Brazil) association with clove (CLO) and cinnamon (CIN) EOs (Mundo dos Óleos^®^, Brasília, Distrito Federal, Brazil). Edible coatings added with sodium alginate (SA) (Himedia^®^, Mumbai, Maharashtra, India) were also assessed at this first stage to enable comparisons to commonly used coatings. CS solution (*w*/*v*) was tested at concentrations ranging from 1.0% to 5.0% and at SA (*w*/*v*) concentrations ranging from 2.0% to 5.0%. Results showed that 1% and 2% SA, as well as 3% and 4% CS, were the concentrations leading to the best outcomes; thus, they were selected to be used in treatments assessed in the herein-conducted experiment (Table 1). All treatments were assessed in vitro, but only CS treatments were applied to papaya samples. SA was used in the analysis conducted in vitro to enable comparisons to the herein proposed edible coating.

The SA-based coating was formed by keeping the solution at 70 °C for 30 min, and, subsequently, by cooling it to 15 °C [40]. The suspension was heated in water at 70 °C, for approximately 15 min, under constant stirring, until the gel was formed to prepare the CS coating. Then, it was left to rest until it reached room temperature. EOs were added to the coatings at the proportion of 2 mL/L of coating. The flowchart of the process is summarized in Figure 1.

Previously washed, sanitized, and drained papayas were immersed once in approximately 700 mL (amount pre-defined as enough to cover the whole fruit) of the coating solution, for 2 min. Then, fruits were drained, placed in containers, and stored at 25 °C, for 12 days. Experimental day 1 refers to the first day of the fresh product right after treatment preparation.

### 2.3. Concentration Tests Conducted In Vitro with EOs Presenting Antimicrobial Effect

*E. coli* culture was obtained from the stock of cultures kept in the Microbiology laboratory of the Federal University of Espírito Santo. *Salmonella* Enteritidis ATCC 13076 culture was obtained from the stock of cultures kept by the Food Hygiene and Microbiology laboratory of the Federal University of Viçosa. Fungal cultures were isolated from contaminated fruits.

The antimicrobial effects of EO-incorporated coatings were assessed based on using inoculation with molds (*Colletotrichum gloeosporioides* and *Penicillium* spp.) and bacteria (*E. coli* ATCC 25922 and *Salmonella* Enteritidis ATCC 13076), in compliance with the methodology adapted by Silvestri et al. [46]. Fungal cultures were standardized at concentrations of 10^4^ spores/mL, whereas bacterial cultures were standardized at concentrations of 10^6^ CFU/mL. The agar diffusion inhibition technique was applied in compliance with the manufacturer’s instructions, according to which, potato dextrose agar (Himedia^®^, Mumbai, Maharashtra, India) was sterilized, cooled, and distributed in Petri dishes. Inoculation was performed on the entire agar surface, based on using a sterile cotton swab immersed in the prepared suspension.

Sterilized plastic molds were used to make ‘wells’ in the agar; then, 60 µL of each edible coating was inoculated in each cavity. Plates were kept under refrigeration at temperatures ranging from 7 °C to 10 °C, overnight, for antimicrobials’ diffusion purposes; then, they were incubated at 25 °C, for 18 h. After the incubation period was over, inhibition halos were measured (in centimeters) by placing a millimeter ruler on the inverted Petri dish.

### 2.4. Physicochemical Featuring of Papaya Fruits

Papaya fruits’ pH and total soluble solid (TSS) contents were analyzed based on recommendations by the Association of Official Analytical Chemists [47], at storage days 1, 3, 6, 9, and 12; at 25 °C. Fruit pH was measured with pH meter (Tecnopon^®^, mPA210, Piracicaba City, São Paulo State, Brazil), based on using 10 g of samples from each treatment homogenized with 100 mL of distilled water. TSS was determined through refractometry based on using an analogical refractometer (Instrutherm^®^, Freguesia do Ó, São Paulo, Brazil), at 25 °C. Readings were performed based on using three drops of pulp added with 10 g of homogenized sample. Results are expressed in °Brix.

Physiological weight loss (PWL) was calculated based on the difference between the initial papaya weight and its final weight, at each storage time (1, 3, 6, 9, and 12). Results are expressed as mass loss rate based on Equation (1).

(1) $${\mathrm{PWL}}_{n}\left[\%\right]=\frac{W_{0}\;-\;W_{n}}{W_{0}}\;\times \;100$$

wherein PWL_n_ is the physiological weight loss rate at each storage time; W_0_ is the initial sample weight (in g) at the first storage day, and W_n_ is the sample weight measured at each storage time.

### 2.5. Vitamin C and Total Carotenoids Content in Papaya Fruits

Vitamin C was estimated through a 2.6-dichlorophenol-indophenol titration based on using 10 g of each homogenized sample [47].

Total carotenoid extraction was performed according to Rodriguez-Amaya [48], with slight modifications. Approximately 5 g of papaya fruit was added to 60 mL of cooled acetone (divided into three 20 mL volumes). Then, the extract was carefully transferred to a separating funnel, which was previously added with 50 mL of cooled petroleum ether to enable transferring pigments from acetone to petroleum ether. Next, 60 mL of distilled water was added to it for full acetone removal purposes. The water was discarded and subsequently, the extracts were stored in amber glass bottles and read in UV/Vis spectrophotometer (Novainstruments Series 2000-325 A 1000 nm, Piracicaba City, São Paulo State, Brazil), based on using quartz cuvettes at 450 nm absorbance. Finally, total carotenoid content was calculated through Equation (2), wherein absorptivity coefficient (E^1%^_1 cm_) refers to β-carotene for petroleum ether. Total carotenoid content was expressed in µg of β-carotene/100 g of sample.

(2) $$\mathrm{CT}\;(µg\;\times \;g^{-1})=\frac{\mathrm{Abs}\;\times \;\mathrm{Vol}\;\times \;10^{4}}{E_{1\mathrm{cm}}^{1\%}\;\times \;P}$$

wherein CT = total carotenoids; Abs = absorbance at maximum λ; Vol = dilution volume (mL); $E_{1\mathrm{cm}}^{1\%}$ = 2592; P = sample weight (g).

### 2.6. Microbiological Analysis

Analyses conducted during the fruits’ storage time were carried out on the first (Day 1) and last storage days (Day 12). Procedures adopted at this stage were based on the American Public Health Association (APHA) methodology [49]. Molds and yeasts were determined based on inoculating 0.1 mL of dilutions on the dry surface of 2% potato dextrose agar (BDA, Himedia^®^, Mumbai, Maharashtra, India) acidified to pH 3.5. Plates were incubated without an inversion process, at 25 ± 1 °C, for approximately 5 to 7 days. Aerobic mesophiles were determined in compliance with the pour plate method, based on using 1 mL of the previously prepared dilutions; then, molten Standard Agar for Counting (PCA, Kasvi^®^, São José dos Pinhais, Paraná, Brazil) was added to the plates and kept at 45 °C. Subsequently, plates were incubated at 35 °C for 24 h. Aliquots were plated in duplicate; results are expressed in colony forming units per gram (CFU/g).

### 2.7. Experimental Design and Statistical Analysis

The experiment followed a completely randomized design, with three repetitions. Data were subjected to analysis of variance (ANOVA) to investigate the influence of sources of variations in treatment, time, and treatment*time interaction. Tukey’s test was used to analyze differences between treatments, whereas Pearson’s test was applied to correlation analysis, both at 5% probability level, in InfoStat Statistical Software (version 2012, Cordoba National University, Argentina).

## 3. Results

### 3.1. Test Conducted In Vitro

Microbial inhibition provided by clove and cinnamon EOs was tested in vitro. Inhibition halo formation was observed at all formulation concentrations added with EOs (Table 2). Although SA (2% SA + CLO) had recorded a higher *Penicillium* sp. inhibition halo, its value was statistically equal to that observed for the 3% CS + CIN composition. Furthermore, the other treatments with SA were also statistically similar to those of CS. SA-based formulations also presented a higher *C. gloeosporioides* inhibition halo, mainly the 2% SA + CLO treatment. However, the microbial inhibition results recorded for films based on CS were also statistically equal to those recorded for films based on SA.

Overall, CS-based formulations added with clove EO showed slightly larger inhibition halos for *E. coli*, *S. enterica*, *and Colletotrichum gloeosporioides*, than for formulations added with cinnamon EO. On the other hand, *Penicillium* sp. appeared to be more sensitive to the formulation added with cinnamon EO.

By keeping in mind that the current study was aimed at assessing a more natural edible coating, the following stages comprised selecting CS formulations, namely, at 3% added with cinnamon EO (3CS + CIN), at 3% added with clove EO (3CS + CLO), at 4% added with cinnamon EO (4CS + CIN), and at 4% added with clove EO (4CS + CLO). It was conducted to compose the coating used for papaya fruits’ microbiological, physical, and physicochemical assessment.

### 3.2. Physicochemical Analyses 

#### 3.2.1. pH

**Figure 2:**
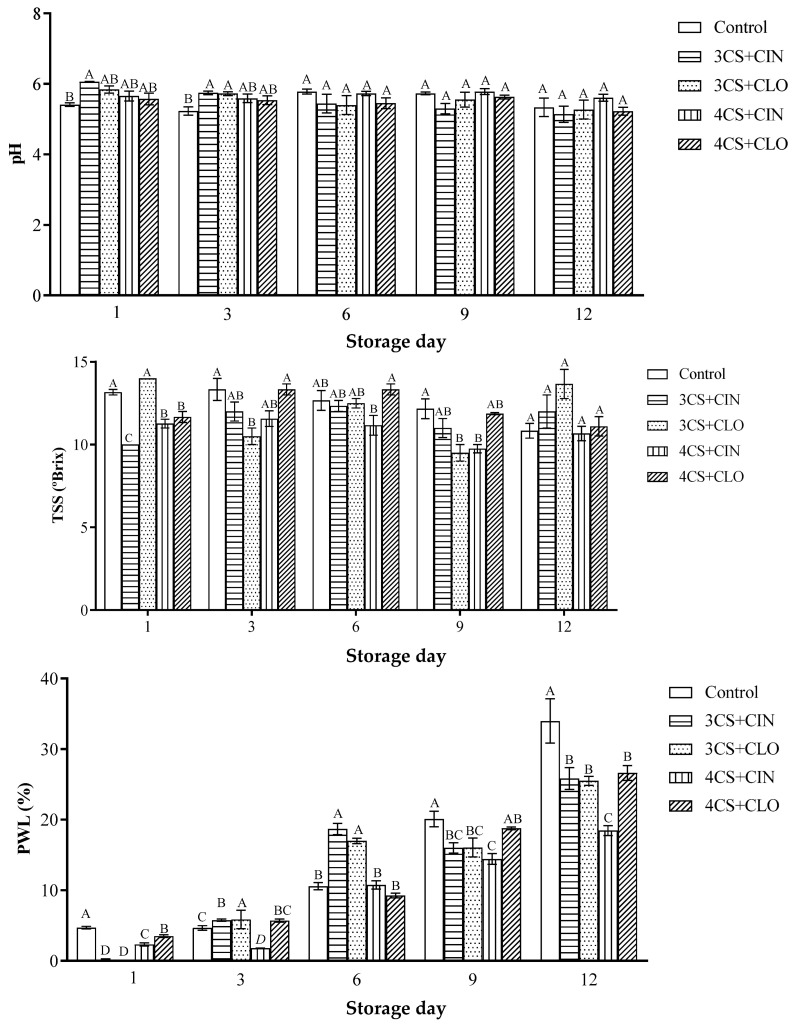
Mean and standard deviation values recorded for pH, total soluble solids (TSS), and physiological weight loss (PWL) of fresh papaya fruits, both uncoated and coated with cassava starch-based coating added with cinnamon or clove essential oil, during storage at 25 °C, for 12 days. Treatments represented by bars showing the same letter, on the same storage day, did not significantly differ from each other in the Tukey test (*p* > 0.05), which was conducted in triplicate.

The recorded pH was statistically equal among the control (5.41 ± 0.09), 3% CS + CLO (5.84 ± 0.17), 4% CS + CLO (5.65 ± 0.25), and 4 % CS + CIN (5.57 ± 0.29) groups (*p*-value > 0.05), on the first storage day. With respect to storage time, papaya fruits coated with 3% CS + CIN recorded higher pH values on the first storage day (Figure 2), and this treatment was the only one differing from the control (*p*-value ≤ 0.05). 

The uncoated papayas recorded values significantly lower than those observed for the coated ones (*p*-value ≤ 0.05), on the third storage day. After the third day, values recorded for this parameter were equal, regardless of coating type. The pH profile of the analyzed samples presented a downward trend over storage time; both the coated and the control papaya fruits recorded statistically equal mean pH values (5.55 ± 0.32) on the 12th storage day (*p*-value > 0.05).

#### 3.2.2. TSS

The present study recorded significant difference in TSS between uncoated papaya fruits (control) and the ones coated with 3% and 4% CS + CIN, as well as with 4% CS + CLO, on the first storage day (Figure 2). These treatments decreased TSS by approximately 16.64% in comparison to values recorded for the uncoated samples. 

Slight variations in TSS happened over time, and a downward trend in its value was observed. The TSS of the uncoated papayas decreased from 13.16 to 10.83 °Brix from the first to the last storage day, respectively, and it represents a reduction by 17.70% in it. Papayas coated with 3% CS + CIN were the only samples recording an increase in this parameter; values ranged from 10 to 12 °Brix, on the first and last storage days. The other treatments also recorded a decrease in TSS by 4.06%, on average (Figure 2). However, this variation was lower than that observed for the uncoated samples, and it indicated that these treatments were effective in preserving this quality attribute.

#### 3.2.3. PWL

Significant physiological weight loss was observed for all the applied treatments and during storage time. The PWL in the uncoated papaya fruits was higher than that observed for the coated ones, on the first storage day (Figure 2).

The PWL tended to increase during storage. Mean weight loss observed for the coated samples on the last storage day reached 24.10%; this value was lower than the one observed for the control treatment, which reached 40.66%.

### 3.3. Vitamin C and Total Carotenoids

**Figure 3:**
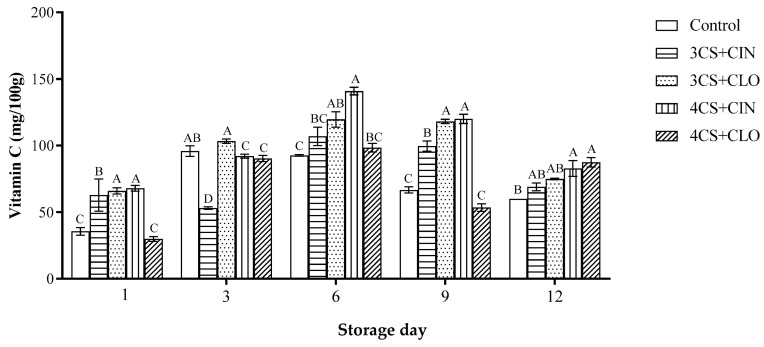

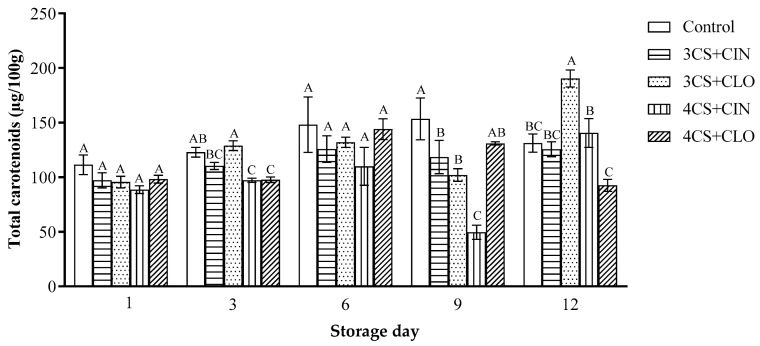
Mean and standard deviation values recorded for vitamin C (mg/100 g) and carotenoid contents (µg/100 g), during the storage of fresh papaya fruits, both uncoated and coated with cassava starch-based coating added with cinnamon or clove essential oil, at 25 °C, for 12 days. Bars with the same letter, on the same storage day, did not significantly differ from each other in the Tukey test (*p* > 0.05), which was conducted in triplicate.

Vitamin C content found in fruits herein subjected to 3CS + CLO, 3CS + CIN, and 4CS + CIN coatings was significantly higher than the one observed for the control, at the first, sixth, and ninth storage days (Figure 3).

Overall, vitamin C content increased up until the sixth storage day, although it decreased after this period. However, coated samples recorded a higher vitamin C retention rate, at the end of storage time (Figure 3). 

Carotenoid content, in its turn, did not show significant difference between uncoated and coated papaya fruits, at the beginning of storage time. Small variations in this parameter were observed during storage; papaya fruits coated with 3CS + CLO recorded the highest value (190.37 µg/100 g) for this parameter, on the 12th storage day. 

### 3.4. Correlation Coefficient of Physicochemical and Nutritional Parameters

The present study has only found positive correlation between total soluble solids and carotenoid content in papaya fruits (Table 3).

### 3.5. Microbiological Load of Papaya Fruits before and after Coating

Different CS concentrations added with EOs have significantly influenced aerobic mesophiles, and mold and yeast growth during storage time (Table 4). Uncoated papaya fruits (control) recorded the highest aerobic mesophile counts on the first storage day, in comparison to the other investigated samples (*p*-value ≤ 0.05). The highest CS concentration (4%) was more efficient in reducing aerobic mesophile count by 1.8 log CFU/g and 2.1 log CFU/g for papaya fruits coated with 4CS + CIN and 4CS + CLO, respectively. Papaya fruits subjected to different edible coatings recorded mean mold and yeast counts statistically equal to each other on the first storage day; it reached 1.48 ± 0.21 log CFU/g, on average. 

The 3CS + CIN coating was the only treatment that did not significantly reduce aerobic mesophilic bacteria count at the end of 12 storage days, in comparison to the first day. Still, the count value recorded for this sample was lower than that of the control sample. With respect to the post-storage yeast and mold counts, no colony was identified in the analyzed samples, at the lowest adopted dilution. 

## 4. Discussion

### 4.1. Inhibition Test Conducted In Vitro

In this work, we evaluate the effect of edible coating against different pathogenic and spoilage microorganisms. The proposal is to demonstrate the potential of edible coating with essential oils to control the presence of these microorganisms.

Souza et al. [23] assessed the antimicrobial effect of CS-based film incorporated with clove and cinnamon EOs. Based on their results, which were similar to the herein observed ones, cinnamon has shown a higher inhibition rate than that of clove EO against *Penicillium commune* and *Eurotium amstelodami.*

Molds are the main cause of deterioration during papaya fruits’ post-harvest period. Anthracnose—caused by *C. gloeosporioides*—is the main disease affecting papaya fruits [7], because lesions caused by it make this product unsuitable for consumption [1,50]. Therefore, spoilage fungi inactivation has been the object of studies aimed at prolonging papaya fruits’ shelf life [7,8,51].

Nevertheless, the incidence of pathogenic bacteria in fruits is a matter of concern since ingesting these microorganisms can lead to severe health issues. Furthermore, food outbreaks have already been recorded due to papaya fruits’ contamination with *Salmonella* sp. [52,53]. According to Pandey et al. [54], cinnamon oils and eugenol and cinnamaldehyde present antimicrobial impacts on Gram-negative and Gram-positive bacteria as *Salmonella enterica*, *E. coli* and *Listeria monocytogenes*. Cinnamon essential oil causes *Salmonella* Enteritidis to generate a significant quantity of reactive oxygen substances (ROS), content of malondialdehyde and protein carbonylation levels, which can cause oxidative damage to cells and irreparable injury to cells [55]. Araújo et al. [56] evaluated the antimicrobial activity of a nanoemulsion of cashew gum and clove essential oil and observed a good action against the pathogenic gastroenteritis species, *Escherichia coli* and *Salmonella enterica*. 

Overall, results have evidenced that the herein proposed substrates were efficient for microbial inhibition purposes.

### 4.2. Physicochemical Parameters of Papaya Fruits 

Fruits undergo physiological and biochemical changes capable of changing their color, texture, aroma, and nutrient content during the ripening process. Therefore, it is essential to control this process to maintain fruit quality and to reduce losses in the post-harvest period [57]. Analytical methods used to determine soluble solids, pH, and carbohydrates, for example, are among the existing ways to monitor the fruit ripening process. These methods play an important part in quality parameters, such as aroma and flavor [6].

Batista et al. [12] did not find a statistically significant difference between pH values recorded for papaya fruits coated with CS (2.5%) and clove EOs. According to the aforementioned authors, maintaining fruits’ chemical features is an interesting strategy because it guarantees products’ natural features. The lower pH observed in stored fruits is associated with sugar metabolism and, consequently, with acid production in the ripening process [7]. Unlike the present study, Mendy et al. [6] observed increased pH in papaya fruits coated with aloe vera; this outcome was associated with delayed fruit ripening.

The pH oscillations resulting from physiological processes were associated with the presence of organic acids, such as malic and citric acid, in the assessed fruits. Reduction in these acids is expected during the post-harvest and storage period of climacteric fruits, such as papaya, due to acids’ consumption (as substrate) for sugar production purposes [8,58,59]. The pH of fruit can increase during storage time due to enzymatic activity and fruit senescence, which in turn leads to reduced acid content in fruits. On the other hand, the maintenance of this parameter, as observed in the present study, has indicated that the proposed treatments were effective in preserving papaya fruits’ quality.

All water-soluble molecules, such as sugar, acids, vitamins, and minerals, are considered soluble solids [14]; however, it is well known that most TSSs are carbohydrates [60]. Moreover, TSS content can change depending on fruits’ genotype and ripening stage [61]. According to Barrera et al. [62], TSS variations can take place due to increased starch metabolism, as well as to monosaccharide and disaccharide production. Consequently, there is a reduction in the amount of astringent compounds and fruits become sweeter at the last ripening stage. Furthermore, the amount of TSS observed in fruits is directly proportional to their weight [14]. Mendy et al. [6] and dos Passos Braga et al. [8] recorded results similar to the ones observed in the present study for coated papaya fruits. According to them, lack of coating favors faster soluble solids’ increase during the ripening process. It happens because edible coatings delay fruits’ respiration and metabolic activity; thus, substrates, such as soluble solids and organic acids, degrade more slowly [14]. Furthermore, coated fruits delay the production of metabolites, such as ethylene [8], as well as modify their internal atmosphere by increasing CO_2_ and decreasing O_2_ levels in them [14].

PWL is an index (expressed as a fruit’s original weight rate) used in the post-harvest period to assess the fruit’s quality. It explains the moisture loss observed on a product’s surface, which, in its turn, leads to fruit wrinkling and deterioration. The presence of biodegradable films on a fruit’s surface features slows down the plant’s respiration, oxidation reaction, and moistness transmission rates [14]. Moreover, applying EOs in combination with coatings can help reducing fruit-weight loss because their lipidic nature reduces fruits’ permeability to water vapor [8]. Storage time increases nutrient consumption and water transpiration rates in fruits. Thus, it can lead to fruit quality loss, i.e., to textural changes and surface shrinkage [14]. Similar results were previously reported by Lata et al. [63] and Zillo et al. [32]. 

The herein investigated coated papaya fruits presented lower weight loss due to the barrier effect provided by the films, since coatings worked as semi-permeable barriers against gas exchanges, solute movement, and moisture. Thus, as for the present study, the application of coatings added with cinnamon and clove EOs avoided moisture transfer by creating a barrier layer on papaya fruits’ surface.

### 4.3. Nutritional Parameters of Papaya Fruits

Fresh fruits are sources of vitamin C, also known as ascorbic acid; this vitamin plays an essential role in reducing oxidative stress in fruits [64]. However, this nutrient is highly unstable; thus, it can suffer losses in the post-harvest period and in the ripening process. Moreover, these losses can increase due to factors, such as inadequate storage, high temperatures, low humidity level, or physical damage to fruits [14,59]. 

Vitamin C values recorded in the present study were slightly higher than those observed by Sousa et al. [44], Mendy et al. [6], and Islam et al. [33], and similar to the ones reported by Batista et al. [12]. However, it is important emphasizing that food composition changes depending on factors, such as variety, assessment method, and production place, among others.

Higher vitamin C retention took place due to both delayed ripening and reduced biological activity during storage time [65]. Similar to the current results, Mendy et al. [6] reported an initial increase in vitamin C, as well as subsequent reduction in it, in papaya fruits coated with aloe vera and stored for 15 days. Sousa et al. [44] also observed similar behavior in ‘Sunrise Solo’ papayas coated with CS added with ginger EO and stored for 12 days. However, different from the results obtained in the present study, vitamin C values recorded for the coated samples were lower than the ones observed for the uncoated samples.

Vitamin C content increases as fruits ripen, but it decreases after a certain time. According to Lata et al. [63], this reduction may be associated with the action of oxidizing enzymes over storage time. The ascorbic acid content increase observed in coated papayas during storage time, as the one observed both in the present and other studies, can be explained by a likely delay in the oxidation process and by slow L-ascorbic acid transformation into dehydroascorbic acid due to the activity of the oxidizing enzyme. On the other hand, variation in vitamin C content over storage time may have happened due to the action of oxidizing enzymes [65]. Therefore, although this parameter was not assessed in the current study, future studies should investigate the presence of enzymes in fruits to help better understand these findings.

Batista et al. [12] did not find a significant difference in carotenoids’ content in papaya fruits coated with CS and clove EO. Carotenoids are unsaturated compounds that can oxidize and isomerize; consequently, they can lead to losses during storage [6]. The increased carotenoid content observed during storage was associated with chlorophyll pigment degradation and with carotenoid synthesis, since immature fruits are greener due to high chlorophyll content, whereas chlorophylls progressively degrade during the fruit ripening process and change their color to a more yellowish shade [63].

Cell walls undergo several changes mediated by enzymes during the fruit ripening process. These changes comprise glycans’ depolymerization, solubilization, and pectins’ depolymerization. Consequently, there is an increase in carbohydrate and total soluble solids content, as well as in chlorophyll degradation and carotenoid synthesis [57]. This dynamic process explains the herein observed correlation between soluble solids and carotenoids. These parameters are linked to flavor and color improvements taking place during ripening, which make fruits more attractive from consumers’ perspectives [57,63]. 

Lipidic component addition to the coating formulation could act as a barrier in the film, as well as reduce fruit permeability and delay the rate of reactions capable of compromising fruit quality [23]. Therefore, the herein-applied coating was effective in maintaining the nutritional compounds over the investigated period.

### 4.4. Microbiological Quality of Papaya Fruits

Edible films and coatings can be used to establish an obstacle involving the product and the environment from microbiological contamination and proliferation, consequently extending a food’s shelf life [14].

Di Pasqua et al. [66] associated the bactericidal effect of components found in EOs with the process inhibiting the production of essential enzymes to microbial metabolism and with structural changes in membrane lipids. Moreover, EOs’ hydrophobic nature helps in disrupting and destabilizing the microbial membrane, and it leads to both intracellular content leakage and cell destruction [27].

High polysaccharide concentrations in coatings can favor lower microbial counts in samples because of the hydrophilic nature of this element, which showed increased interaction with phenolic compounds found in EOs [67]. Dos Passos Braga et al. [8] observed increased *C. gloesporiodes* growth inhibition in vitro at higher polysaccharide concentrations added with mint EO. Zillo et al. [32] also observed antifungal action against *C. gloesporiodes* in papaya fruits coated with rosemary, pepper, and eucalyptus EOs, for nine storage days. The aforementioned authors associated the reduction in *C. gloesporiodes* counts with changes in fungal wall permeability, which affected essential cell activities. Moreover, anthracnose development was less severe in the coated papaya fruits than in the uncoated ones.

Based on another study, papaya fruits’ coating with gum arabic (10%) and ginger EO (2%) also had a potential fungicidal effect [68]. Md Nor and Ding [69] observed a reduction by 2 log CFU/g in mesophiles and by 3 log CFU/g in yeasts, in papaya fruits coated with chitosan and CS. The initial count (2.5 log CFU/g) recorded by Md Nor and Ding [69] on the first storage day was lower than the one observed in the present study. However, the aerobic mesophile count reached 4 log CFU/g at the end of the storage period; this value was higher than the one observed in the present study. It is essential to emphasize that the coating effect depends on the used material type, as well as on the antimicrobial agent selected for the formulation.

Yeast, mold, and bacteria are the agents mainly accounting for microbiological spoilage in fruits. The mesophilic aerobic bacteria population is an indicator of natural microflora and contamination, whereas yeasts and molds are the main fruit spoilage-causing agents due to favorable pH [14].

The herein-mentioned results indicated that coatings added with EOs presented antimicrobial activity. This effect was associated with phenolic compounds observed in clove and cinnamon oils, such as eugenol and cinnamaldehyde. It is possible to suggest that the higher amount of polysaccharides found in the edible coating has favored EOs’ interaction with the fruit, as well as improved their biocidal effect.

However, no significant impact on this parameter was reported in the present study, likely because of the herein-adopted short storage time. Fruit storage has serious limitations that leads to fast deterioration and high decay rates. Microbial contaminations are directly linked to the post-harvest deterioration of papaya fruits, besides being the main cause of issues observed at this stage; they reduce fruits’ shelf life, and make them unsuitable for consumption within a short amount of time. However, an aspect important to emphasize is that the application of the edible coating is capable of reducing microorganism development in coated fruits and prolonging the shelf life [14].

## 5. Conclusions

CS coatings added with clove and cinnamon EOs were efficient in delaying processes capable of reducing papaya fruits’ quality in the post-harvest period, such as physiological weight loss, in addition to improving fruits’ microbiological parameters. The reduced counts recorded for aerobic mesophiles, yeasts, and molds can be explained by EOs’ antimicrobial effect. The 4CS + CIN and 4CS + CLO coatings were the treatments recording the best microbiological reduction results. Thus, 4% CS coatings added with clove and cinnamon EOs can extend the shelf life of papaya fruits; consequently, they can be used as a microbiological control strategy to improve fruit quality in the post-harvest period. The herein-assessed storage time was not enough to cause significant variations in the analyzed physical–chemical parameters.

EOs’ properties and edible coatings’ effectiveness have indicated the satisfactory potential of this association to increase papaya fruits’ shelf life. However, future studies should be conducted with longer storage times, and sensory tests should be carried out to ensure that the investigated product is well accepted by consumers.

## Figures and Tables

**Table 1:** Treatments assessed in the present experiment.

Treatments	Coatings	Description	References
Test in vitro	1% SA + CLO	Sodium alginate coating at 1% with clove essential oil	[38,39,40,41,42]
	1% SA + CIN	Sodium alginate coating at 1% with cinnamon essential oil	
	2% SA + CLO	Sodium alginate coating at 2% with clove essential oil	
	2% SA + CIN	Sodium alginate coating at 2% with cinnamon essential oil	
Control	None	Uncoated papayas	-
3CS + CLO	3% CS + CLO	Cassava starch coating at 3% with clove essential oil	[43,44,45,46]
3CS + CIN	3% CS + CIN	Cassava starch coating at 3% with cinnamon essential oil	
4CS + CLO	4% CS + CLO	Cassava starch coating at 4% with clove essential oil	
4CS + CIN	4% CS + CIN	Cassava starch coating at 4% with cinnamon essential oil	

SA: sodium alginate; CS: cassava starch; CLO: clove essential oil; CIN: cinnamon essential oil.

**Table 2:** Mean diameter (cm) of microbial inhibition halo after the application of different formulations.

Coatings	Microorganisms			
	*E. coli*	*S. enterica* Enteritidis	*Penicillium* sp.	*Colletotrichum gloeosporioides*
1% SA + CLO	4.66 ± 0.57 ^ab^	4.50 ± 1.32 ^abc^	3.33 ± 0.57 ^cd^	1.66 ± 0.58 ^ab^
1% SA + CIN	4.00 ± 1.73 ^abc^	5.16 ± 0.28 ^a^	3.50 ± 0.50 ^bcd^	1.46 ± 0.15 ^bc^
2% SA + CLO	4.83 ± 0.57 ^a^	4.83 ± 0.57 ^ab^	5.33 ± 0.76 ^a^	2.20 ± 0.78 ^a^
2% SA + CIN	4.00 ± 1.00 ^abc^	3.66 ± 0.73 ^bc^	4.33 ± 0.76 ^abc^	1.43 ± 0.23 ^bc^
3% CS + CLO	3.00 ± 1.00 ^bcd^	3.33 ± 0.28 ^dc^	3.10 ± 0.85 ^cd^	1.10 ± 0.24 ^bc^
3% CS + CIN	2.50 ± 0.86 ^dc^	3.16 ± 1.25 ^dc^	4.93 ± 0.90 ^ab^	0.70 ± 0.11 ^c^
4% CS + CLO	2.26 ± 0.25 ^cd^	3.50 ± 0.50 ^bcd^	2.73 ± 0.92 ^d^	1.30 ± 0.30 ^bc^
4% CS + CIN	2.16 ± 0.76 ^d^	2.16 ± 0.28 ^d^	2.66 ± 1.15 ^d^	0.80 ± 0.16 ^c^

Means and standard deviation followed by the same letter in the same column did not statistically differ from each other in Tukey’s test (*p*-value > 0.05). SA: sodium alginate; CS: cassava starch; CLO: clove essential oil; CIN: cinnamon essential oil.

**Table 3:** Correlation coefficient between papaya fruits’ physicochemical parameters after the application of different coating types and storage at 25 °C.

	pH	TSS	Total Carotenoids	PWL	Vitamin C
pH		−0.0965	−0.0616	−0.3958	−0.00772
TSS			0.4426 *	−0.1681	−0.08013
Total carotenoids				0.1186	−0.04592
PWL					0.11133

* Significant at 5% probability level; TSS: total soluble solid; PWL: physiological weight loss.

**Table 4:** Mean and standard deviation of aerobic mesophilic bacteria, mold, and yeast count (log CFU/g) after the application of different edible coating types on whole papaya fruits stored at 25 °C, for 12 days.

Coating	Aerobic Mesophilic Bacteria (CFU/g)		Molds and Yeasts (CFU/g)	
	Day 1	Day 12	Day 1	Day 12
Control	3.16 ± 0.21 ^aA^	3.07 ± 0.09 ^aA^	3.43 ± 0.29 ^aA^	1.82 ± 0.13 ^bA^
3% CS + CLO	2.34 ± 0.17 ^aB^	n.d.	1.70 ± 0.30 ^aB^	n.d.
3% CS + CIN	2.29 ± 0.54 ^aB^	1.19 ± 0.60 ^aB^	1.66 ± 0.33 ^aB^	n.d.
4% CS + CLO	1.06 ± 0.07 ^aC^	n.d.	1.23 ± 0.09 ^aB^	n.d.
4% CS + CIN	1.03 ± 0.03 ^aC^	n.d.	1.33 ± 0.12 ^aB^	n.d.

Means followed by the same lowercase letter on the same line did not significantly differ from each other in the *t*-test (*p*-value > 0.05). Means followed by the same uppercase letter in the same column did not significantly differ from each other in Tukey’s test (*p*-value > 0.05). CS: cassava starch; CLO: clove essential oil; CIN: cinnamon essential oil; n.d.: not detectable at the lowest dilution performed during analysis.

## Data Availability

Further inquiries can be directed to the corresponding author(s).

## References

[1] Singh S.P., Sudhakar Rao D.V. (2011). Papaya (*Carica papaya* L.). Postharvest Biol. Technol. Trop. Subtrop. Fruits.

[2] (2021). FAO Major Tropical Fruits: Preliminary Results 2020.

[3] Gomes B.L., Fabi J.P., Purgatto E. (2016). Cold Storage Affects the Volatile Profile and Expression of a Putative Linalool Synthase of Papaya Fruit. Food Res. Int..

[4] Ming R., Hou S., Feng Y., Yu Q., Dionne-Laporte A., Saw J.H., Senin P., Wang W., Ly B.V., Lewis K.L.T. (2008). The Draft Genome of the Transgenic Tropical Fruit Tree Papaya (*Carica papaya* Linnaeus). Nature.

[5] Huerta-Ocampo J.Á., Osuna-Castro J.A., Lino-López G.J., Barrera-Pacheco A., Mendoza-Hernández G., De León-Rodríguez A., Barba de la Rosa A.P. (2012). Proteomic Analysis of Differentially Accumulated Proteins during Ripening and in Response to 1-MCP in Papaya Fruit. J. Proteom..

[6] Mendy T.K., Misran A., Mahmud T.M.M., Ismail S.I. (2019). Application of Aloe Vera Coating Delays Ripening and Extend the Shelf Life of Papaya Fruit. Sci. Hortic..

[7] Mendy T.K., Misran A., Mahmud T.M.M., Ismail S.I. (2019). Antifungal Properties of Aloe Vera through in Vitro and in Vivo Screening against Postharvest Pathogens of Papaya Fruit. Sci. Hortic..

[8] dos Passos Braga S., Lundgren G.A., Macedo S.A., Tavares J.F., dos Santos Vieira W.A., Câmara M.P.S., de Souza E.L. (2019). Application of Coatings Formed by Chitosan and Mentha Essential Oils to Control Anthracnose Caused by Colletotrichum Gloesporioides and C. Brevisporum in Papaya (*Carica papaya* L.) Fruit. Int. J. Biol. Macromol..

[9] Maringgal B., Hashim N., Mohamed Amin Tawakkal I.S., Muda Mohamed M.T. (2020). Recent Advance in Edible Coating and Its Effect on Fresh/Fresh-Cut Fruits Quality. Trends Food Sci. Technol..

[10] de Oliveira Filho J.G., Silva G.d.C., Oldoni F.C.A., Miranda M., Florencio C., de Oliveira R.M.D., Gomes M.d.P., Ferreira M.D. (2022). Edible Coating Based on Carnauba Wax Nanoemulsion and Cymbopogon Martinii Essential Oil on Papaya Postharvest Preservation. Coatings.

[11] Jarzębski M., Smułek W., Siejak P., Rezler R., Pawlicz J., Trzeciak T., Jarzębska M., Majchrzak O., Kaczorek E., Kazemian P. (2021). Aesculus *Hippocastanum* L. as a Stabilizer in Hemp Seed Oil Nanoemulsions for Potential Biomedical and Food Applications. Int. J. Mol. Sci..

[12] de Vasconcellos Santos Batista D., Reis R.C., Almeida J.M., Rezende B., Bragança C.A.D., da Silva F. (2020). Edible Coatings in Post-Harvest Papaya: Impact on Physical–Chemical and Sensory Characteristics. J. Food Sci. Technol..

[13] Mosa M.A., Youssef K., Hamed S.F., Hashim A.F. (2023). Antifungal Activity of Eco-Safe Nanoemulsions Based on Nigella Sativa Oil against Penicillium Verrucosum Infecting Maize Seeds: Biochemical and Physiological Traits. Front. Microbiol..

[14] Chavan P., Lata K., Kaur T., Rezek Jambrak A., Sharma S., Roy S., Sinhmar A., Thory R., Pal Singh G., Aayush K. (2023). Recent Advances in the Preservation of Postharvest Fruits Using Edible Films and Coatings: A Comprehensive Review. Food Chem..

[15] Han B., Chen P., Guo J., Yu H., Zhong S., Li D., Liu C., Feng Z., Jiang B. (2023). A Novel Intelligent Indicator Film: Preparation, Characterization, and Application. Molecules.

[16] González A., Gastelú G., Barrera G.N., Ribotta P.D., Álvarez Igarzabal C.I. (2019). Preparation and Characterization of Soy Protein Films Reinforced with Cellulose Nanofibers Obtained from Soybean By-Products. Food Hydrocoll..

[17] Hu Y., Shi L., Ren Z., Hao G., Chen J., Weng W. (2021). Characterization of Emulsion Films Prepared from Soy Protein Isolate at Different Preheating Temperatures. J. Food Eng..

[18] Zhou Y., Wu X., Chen J., He J. (2021). Effects of Cinnamon Essential Oil on the Physical, Mechanical, Structural and Thermal Properties of Cassava Starch-Based Edible Films. Int. J. Biol. Macromol..

[19] Bester A.U., Carvalho I.R., Da Silva J.A.G., Hutra D.J., Moura N.B., Lautenchleger F., Loro M.V. (2021). Three Decades of Cassava Cultivation in Brazil: Potentialities and Perspectives. Rev. Colomb. Cienc. Hortícolas.

[20] Yousuf B., Qadri O.S. (2020). Preservation of Fresh-Cut Fruits and Vegetables by Edible Coatings. Fresh-Cut Fruits and Vegetables Technologies and Mechanisms for Safety Control.

[21] Pérez-Vergara L.D., Cifuentes M.T., Franco A.P., Pérez-Cervera C.E., Andrade-Pizarro R.D. (2020). Development and Characterization of Edible Films Based on Native Cassava Starch, Beeswax, and Propolis. NFS J..

[22] Lim W.S., Ock S.Y., Park G.D., Lee I.W., Lee M.H., Park H.J. (2020). Heat-Sealing Property of Cassava Starch Film Plasticized with Glycerol and Sorbitol. Food Packag. Shelf. Life.

[23] Souza A.C., Goto G.E.O., Mainardi J.A., Coelho A.C.V., Tadini C.C. (2013). Cassava Starch Composite Films Incorporated with Cinnamon Essential Oil: Antimicrobial Activity, Microstructure, Mechanical and Barrier Properties. LWT Food Sci. Technol..

[24] Chisenga S.M., Workneh T.S., Bultosa G., Alimi B.A. (2019). Progress in Research and Applications of Cassava Flour and Starch: A Review. J. Food Sci. Technol..

[25] Antunes M.D., Gago C.M., Cavaco A.M., Miguel M.G. (2012). Edible coatings enriched with essential oils and their compounds for fresh and fresh-cut fruit. Recent Patents Food Nutr. Agric..

[26] Suhag R., Kumar N., Petkoska A.T., Upadhyay A. (2020). Film formation and deposition methods of edible coating on food products: A review. Food Res. Int..

[27] Xu T., Gao C., Feng X., Huang M., Yang Y., Shen X., Tang X. (2019). Cinnamon and clove essential oils to improve physical, thermal and antimicrobial properties of chitosan-gum arabic polyelectrolyte complexed films. Carbohydr. Polym..

[28] El Asbahani A., Miladi K., Badri W., Sala M., Aït Addi E.H., Casabianca H., El Mousadik A., Hartmann D., Jilale A., Renaud F.N.R. (2015). Essential oils: From extraction to encapsulation. Int. J. Pharm..

[29] Prakash A., Baskaran R., Paramasivam N., Vadivel V. (2018). Essential oil based nanoemulsions to improve the microbial quality of minimally processed fruits and vegetables: A review. Food Res. Int..

[30] Delshadi R., Bahrami A., Tafti A.G., Barba F.J., Williams L.L. (2020). Micro and nano-encapsulation of vegetable and essential oils to develop functional food products with improved nutritional profiles. Trends Food Sci. Technol..

[31] Oliveira P.D.L., de Oliveira K.Á.R., Vieira W.A.D.S., Câmara M.P.S., de Souza E.L. (2018). Control of anthracnose caused by Colletotrichum species in guava, mango and papaya using synergistic combinations of chitosan and Cymbopogon citratus (D.C. ex Nees) Stapf. essential oil. Int. J. Food Microbiol..

[32] Zillo R.R., da Silva P.P.M., de Oliveira J., da Glória E.M., Spoto M.H.F. (2018). Carboxymethylcellulose coating associated with essential oil can increase papaya shelf life. Sci. Hortic..

[33] Islam M.Z., Saha T., Monalisa K., Hoque M.M. (2019). Effect of starch edible coating on drying characteristics and antioxidant properties of papaya. J. Food Meas. Charact..

[34] Castro M., Mantuano M.-I., Coloma J.-L., Santacruz S. (2017). Utilisation of Cassava Starch Edible Films Containing Salicylic Acid on Papaya (*Carica papaya* L.) Preservation. Rev. Politécnica.

[35] Holsbach F.M.S., Pizato S., Fonteles N.T., De Souza P.D., Pinedo R.A., Cortez-Vega W.R., Universidade Federal da Grande Dourados (2019). Avaliação da vida útil de mamão formosa (*Carica papaya* L.) minimamente processado utilizando coberturas de amido de mandioca e óleo essencial de cravo. J. Bioenergy Food Sci..

[36] Murmu S.B., Mishra H.N. (2018). The effect of edible coating based on Arabic gum, sodium caseinate and essential oil of cinnamon and lemon grass on guava. Food Chem..

[37] Wang W., Zhang Y., Yang Z., He Q. (2021). Effects of incorporation with clove (*Eugenia caryophyllata*) essential oil (CEO) on overall performance of chitosan as active coating. Int. J. Biol. Macromol..

[38] Silva D.A., Oliveira J.K., Santos C.M., Bery C.C.S., Castro A.A., Santos J.A.B. (2014). The Use of Sodium Alginate-Based Coating and Cellulose Acetate in Papaya Post-Harvest Preservation. Acta Scientiarum. Technol..

[39] Farina V., Passafiume R., Tinebra I., Scuderi D., Saletta F., Gugliuzza G., Gallotta A., Sortino G. (2020). Postharvest Application of Aloe Vera Gel-Based Edible Coating to Improve the Quality and Storage Stability of Fresh-Cut Papaya. J. Food Qual..

[40] Rojas-Graü M.A., Raybaudi-Massilia R.M., Soliva-Fortuny R.C., Avena-Bustillos R.J., McHugh T.H., Martín-Belloso O. (2007). Apple Puree-Alginate Edible Coating as Carrier of Antimicrobial Agents to Prolong Shelf-Life of Fresh-Cut Apples. Postharvest. Biol. Technol..

[41] Rangel-Marrón M., Mani-López E., Palou E., López-Malo A. (2019). Effects of Alginate-Glycerol-Citric Acid Concentrations on Selected Physical, Mechanical, and Barrier Properties of Papaya Puree-Based Edible Films and Coatings, as Evaluated by Response Surface Methodology. LWT Food Sci. Technol..

[42] Tavassoli-Kafrani E., Shekarchizadeh H., Masoudpour-Behabadi M. (2016). Development of Edible Films and Coatings from Alginates and Carrageenans. Carbohydr. Polym..

[43] Castricini A., Coneglian R.C.C., Deliza R. (2012). Starch Edible Coating of Papaya: Effect on Sensory Characteristics. Food Sci. Technol..

[44] Da Mota Sousa F.D.A.R., Martins Véras M.L., De Melo Silva S. (2021). Postharvest Conservation of ‘Sunrise Solo’ Papaya under Cassava Starch Coatings Added with Ginger Essential Oil. Comun. Sci..

[45] Praseptiangga D., Utami R., Khasanah L.U., Evirananda I.P. (2017). Kawiji Effect of Cassava Starch-Based Edible Coating Incorporated with Lemongrass Essential Oil on the Quality of Papaya MJ9. IOP Conf. Ser. Mater. Sci. Eng..

[46] Silvestri J.D.F., Paroul N., Czyewski E., Lerin L., Rotava I., Cansian R.L., Mossi A., Toniazzo G., De Oliveira D., Treichel H. (2010). Chemical Composition and Antioxidant and Antibacterial Activities of Clove Essential Oil (*Eugenia caryophyllata* Thunb). Rev. Ceres.

[47] Horwitz W., Latimer G. (2005). AOAC Official Methods of Analysis of AOAC International.

[48] Rodriguez-Amaya D.B. (2001). A Guide to Carotenoid Analysis in Foods.

[49] Downes F.P., Ito K. (2001). Compendium of Methods for The Microbiological Examination of Foods.

[50] Paull R.E., Oliveira J.G. (2020). Tropical Fruits: Papayas. Controlled and Modified Atmospheres for Fresh and Fresh-Cut Produce.

[51] Vilaplana R., Chicaiza G., Vaca C., Valencia-Chamorro S. (2020). Combination of hot water treatment and chitosan coating to control anthracnose in papaya (*Carica papaya* L.) during the postharvest period. Crop Prot..

[52] Whitney B.M., McClure M., Hassan R., Pomeroy M., Seelman S.L., Singleton L.N., Blessington T., Hardy C., Blankenship J., Pereira E. (2021). A Series of Papaya-Associated Salmonella Illness Outbreak Investigations in 2017 and 2019: A Focus on Traceback, Laboratory, and Collaborative Efforts. J. Food Prot..

[53] CDC Salmonella Outbreak Linked to Papayas. https://www.cdc.gov/media/releases/2019/s0628-salmonella-outbreak-papayas.html.

[54] Pandey V.K., Islam R.U., Shams R., Dar A.H. (2022). A Comprehensive Review on the Application of Essential Oils as Bioactive Compounds in Nano-Emulsion Based Edible Coatings of Fruits and Vegetables. Appl. Food Res..

[55] Zhang Z., Zhao Y., Chen X., Li W., Li W., Du J., Wang L. (2022). Effects of Cinnamon Essential Oil on Oxidative Damage and Outer Membrane Protein Genes of Salmonella Enteritidis Cells. Foods.

[56] Araujo T.D.S., da Costa J.M.A.R., de Oliveira Silva Ribeiro F., de Jesus Oliveira A.C., do Nascimento Dias J., de Araujo A.R., Barros A.B., da Paixão Brito M., de Oliveira T.M., de Almeida M.P. (2021). Nanoemulsion of Cashew Gum and Clove Essential Oil (Ocimum Gratissimum Linn) Potentiating Antioxidant and Antimicrobial Activity. Int. J. Biol. Macromol..

[57] Alós E., Rodrigo M.J., Zacarias L. (2019). Ripening and Senescence. Postharvest Physiology and Biochemistry of Fruits and Vegetables.

[58] Tabassum N., Khan M.A. (2020). Modified atmosphere packaging of fresh-cut papaya using alginate based edible coating: Quality evaluation and shelf life study. Sci. Hortic..

[59] Prasad K., Jacob S., Siddiqui M.W. (2018). Fruit Maturity, Harvesting, and Quality Standards. Preharvest Modulation of Postharvest Fruit and Vegetable Quality.

[60] Khandpur P., Gogate P.R. (2015). Effect of novel ultrasound based processing on the nutrition quality of different fruit and vegetable juices. Ultrason. Sonochem..

[61] Bhat R., Goh K.M. (2017). Sonication treatment convalesce the overall quality of hand-pressed strawberry juice. Food Chem..

[62] Barrera E., Gil J., Restrepo A., Mosquera K., Durango D. (2015). A Coating of Chitosan and Propolis Extract for the Postharvest Treatment of Papaya (*Carica papaya* L. Cv. Hawaiiana). Rev. Fac. Nac. Agron. Medellin..

[63] Lata D., Aftab M.A., Homa F., Ahmad S., Siddiqui M.W. (2018). Effect of eco-safe compounds on postharvest quality preservation of papaya (*Carica papaya* L.). Acta Physiol. Plant..

[64] Khaliq G., Mohamed M.T.M., Ali A., Ding P., Ghazali H.M. (2015). Effect of gum arabic coating combined with calcium chloride on physico-chemical and qualitative properties of mango (*Mangifera indica* L.) fruit during low temperature storage. Sci. Hortic..

[65] Hazarika T.K., Lalthanpuii, Mandal D. (2017). Influence of Edible Coatings on Physico-Chemical Characteristics and Shelf-Life of Papaya (*Carica papaya*) Fruits during Ambient Storage. Indian J. Agric. Sci..

[66] Di Pasqua R., Betts G., Hoskins N., Edwards M., Ercolini D., Mauriello G. (2007). Membrane Toxicity of Antimicrobial Compounds from Essential Oils. J. Agric. Food Chem..

[67] Chollakup R., Pongburoos S., Boonsong W., Khanoonkon N., Kongsin K., Sothornvit R., Sukyai P., Sukatta U., Harnkarnsujarit N. (2020). Antioxidant and Antibacterial Activities of Cassava Starch and Whey Protein Blend Films Containing Rambutan Peel Extract and Cinnamon Oil for Active Packaging. LWT Food Sci. Technol..

[68] Ali A., Hei G.K., Keat Y.W. (2016). Efficacy of ginger oil and extract combined with gum arabic on anthracnose and quality of papaya fruit during cold storage. J. Food Sci. Technol..

[69] Md Nor S., Ding P. (2020). Trends and Advances in Edible Biopolymer Coating for Tropical Fruit: A Review. Food Res. Int..

